# Trapped radical behavior of electron beam irradiated polytetrafluoroethylene fine powder at various temperatures

**DOI:** 10.1038/s41598-021-90462-6

**Published:** 2021-05-25

**Authors:** Akihiro Oshima, Hiroto Horiuchi, Atsushi Nakamura, Shun Kobayashi, Ayana Terui, Ayano Mino, Ryoya Shimura, Masakazu Washio

**Affiliations:** 1 Graduate School of Engineering, Osaka University, 2-1 Yamadaoka, Suita, Osaka 565-0871 Japan; 2 The Institute of Scientific and Industrial Research, Osaka University, 8-1 Mihogaoka, Ibaraki, Osaka 567-0047 Japan; 3 Waseda Research Institute for Science and Engineering, Waseda University, 3-4-1, Okubo, Shinjuku, Tokyo 169-8555 Japan

**Keywords:** Materials science, Soft materials, Polymers, Polymer chemistry

## Abstract

Polytetrafluoroethylene (PTFE) fine powder with 93% crystallinity was irradiated by an electron beam (EB) at various temperatures under a nitrogen atmosphere. Trapped free radicals in PTFE were studied using electron spin resonance (ESR) spectroscopy. The observed spectra of the samples exposed to air after irradiation at various temperatures showed asymmetrical signals, which are middle-chain type peroxide macroradicals derived from fluoroalkyl radicals. The radical yields at each irradiation temperature increased with increasing absorbed dose, and eventually saturated. The higher irradiation temperature resulted in higher radical yields when compared at the same exposed dose. Furthermore, the G-value of the radicals (G(R·)) increases with increasing irradiation temperatures corresponding to each relaxation and transition temperature. It is concluded that the chain reaction by the fluorine extraction from the main chain due to the end-chain radical generated via β-scission after dissociative electron attachment (DEA) is enhanced by the synergistic effect of heat and radiation.

## Introduction

In a study on the temperature dependence of irradiation effects on polymer materials some interesting phenomena were reported. For example, the cross-linking efficiency of polyethylene increased by approximately 20% in the molten state irradiation compared with room temperature (RT) irradiation^1,2^. Polystyrene, which is mainly taken place the cross-linking reaction at RT irradiation, has a predominant chain scission reaction at irradiation above the glass transition (Tg) temperature^2–4^. The radiation-induced chain scission of PTFE takes place over a wide range of temperatures below the temperature range (596–623 K) in which the melting of PTFE crystallites occurs^2,5–7^ and the scission efficiency significantly increases with increasing irradiation temperature^2,6^, although crosslinking takes place at a molten state temperature of approximately 608 K^2,8–13^. This means that the radiation-induced cross-linking or scission of polymers strongly depends on the irradiation temperature and is affected by the molecular motion of each polymer^2^.

According to a study of radical formation in PTFE, when irradiation at either low temperature or RT under an oxygen-free atmosphere is carried out, two types of radicals are reported as fluoroalkyl (–CF_2_–${\dot{\text{C}}}$F–CF_2_–) and end-chain radical (–CF_2_–${\dot{\text{C}}}$F_2_)^14–16^. These radicals are trapped in the crystalline phase and are rather stable under vacuum. As the hyperfine splitting of F-atoms has a state of anisotropy and a relatively large value, the ESR spectrum shows their specific broad patterns originating from these radical species. It has already reported that both fluoroalkyl and end-chain radicals react easily with oxygen and convert to the peroxide macroradicals^16–18^. That is, the former one is an fluoroalkyl-based chain type peroxide macroradical –CF_2_–CF(OO·)–CF_2_–: middle-chain) and the latter one is an end-chain type peroxide macroradical (–CF_2_–CF_2_OO·: end-chain), which are mainly induced in the crystalline region^16,17^.

Although the lifetime (half-decay time) of trapped peroxide macroradicals is estimated to be 1000 h when stored at 297 K under an oxygen-free atmosphere, radicals are gradually annihilated by heat relaxation^17,18^.

In this study, the effect of irradiation temperature on the radiation-induced scission reaction of PTFE is discussed from the viewpoint of trapped radical behavior.

## Materials and methods

Commercially available homopolymerized PTFE fine powders (POLYFLON-PTFE, ρ: 2.17) with an average particle size of ca 500 µm were supplied by Daikin Industries Ltd. The molecular weight of PTFE (1.73 × 10^6^ Da) was determined from the heat of crystallization of PTFE using a differential scanning calorimeter (DSC: Perkin Elmer: PYRIS DIAMOND DSC)^6^. The degree of crystallinity 93% was determined with X-ray diffractometry (XRD; scattering range *2θ* = 10°–25° for crystallinity calculation with peak area method) and the mean size of the PTFE crystallites was 25.6 nm (Miller's indices hkl = 100) using Debye–Scherrer equation. PTFE powders were molded into a 1 mm diameter string (apparent density of 0.85 g cm^−3^, see Supplementary Information).

The electron beam (EB) irradiation was carried out at various temperatures ranging from 298 to 523 K under nitrogen atmosphere using a 200 kV electron accelerator (CURETRON, NHV corp., maximum electron energy: 200 keV, maximum electron current: 20 mA, effective irradiation width: 15 cm) installed at Waseda Research Institute for Science and Engineering, Waseda University. PTFE strings were placed in an irradiation vessel (External shape: 210^L^ × 210^ W^ × 53^ T^ mm^3^, see Supplementary Information) with a heating device, and then heated to the desired temperature (± 5 K) under nitrogen atmosphere. From the Monte Carlo simulation code EGS5^19,20^, the penetration depth of 200 keV-EB with an apparent density of 0.85 is approximately 500 μm. Thus, in the present experiment, it is not irradiated to the part of PTFE string. The condition of electron equilibrium is fulfilled in this experiment of EB irradiation of PTFE samples. The 200 keV-EB irradiation was done stepwise with a dose of 15 kGy per pass (acceleration voltage: 200 kV, electron current: 0.5 mA, dose rate: 5 kGy s^−1^, see Supplementary Information), and then the irradiated PTFE was exposed to air at 298 K within 5 min and stored at 298 K under atmosphere.

After EB irradiation, the sample was immediately exposed to air, and then the samples (200.0 ± 5.0 mg) inserted into a 5-mm diameter quartz tube for ESR spectroscopy. Trapped free radicals were analyzed using an X-band ESR spectrometer (JES-RE1X, JEOL) at 297 K after irradiation within 1 h. The ESR measurement parameters, namely the microwave frequency and power, the sweep range of the magnetic field, the field modulation width of 100 kHz, and the time constant were set to 9.43 GHz (wavelength: 3.18 cm), 40–100 μW, 333.0 ± 15 mT, 0.2 mT, and 0.1 s, respectively. The total spin intensity was calculated by double integrating the spectrum and normalized using the instrument parameters. The radical yields (spin g^−1^) were determined from the spin intensity after calibration with DPPH (2,2-Diphenyl-1-picrylhydrazyl, 1.58 × 10^23^ spin g^−1^). From the irradiation range of 200 keV-EB mention above, only a part of the PTFE string was irradiating. Although, in order to calculate the absolute value of the trapped radical yields, it is necessary to perform the irradiation area correction, the radical yields were normalized per total weight without the area correction in this experiment.

## Results and discussion

**Figure 1:**
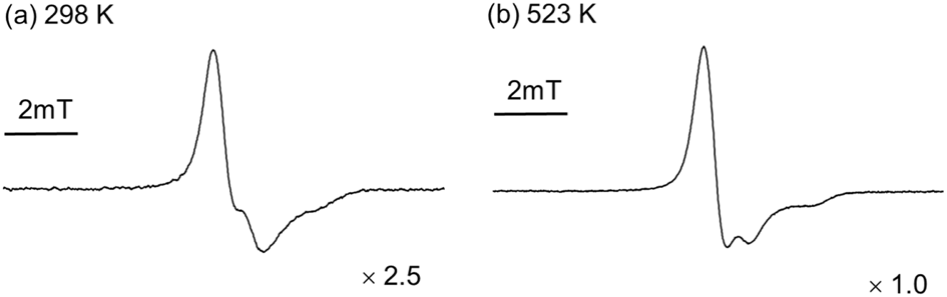
ESR spectra of trapped free radicals in PTFE measured at 297 K after EB irradiation within 1 h. (**a**) Irradiation carried out with a dose of 15 kGy at 298 K (radical yield: RY = 5.24 × 10^16^ spin g^−1^) and (**b**) 523 K (RY = 1.37 × 10^17^ spin g^−1^) under nitrogen atmosphere, followed by exposure to air at 298 K within 5 min and stored at 298 K under atmosphere until measurement.

Figure 1 shows the ESR spectra of the trapped free radicals in PTFE measured at 297 K within 1 h after irradiation. EB irradiation was carried out at a dose of 15 kGy at 298 K (A) and 523 K (B) under a nitrogen atmosphere and then the sample exposed to air at 298 K for 5 min. The observed spectra showed asymmetric signals, and they were assigned as peroxide macroradicals. These are a superposition of major asymmetric signals assigned from the middle-chain type peroxide macroradical and minor symmetric signals from the end-chain type peroxide macroradical, as already reported^16–18^. It was noticed that the signal intensity was more intense when the irradiation temperature was higher.

It was reported previously that the radical decay of PTFE accelerated with heat treatment^17,18^, the thermal stability of the fluoroalkyl and chain-end radicals are different, and the chain-end radicals were decayed faster^18^. It is suggested that the difference in the spectral shape in Fig. 1 would reflect the difference between the amount of the trapped middle-chain and chain-end types peroxide macroradicals. Furthermore, the results of the improved radical yields at the higher irradiation temperature observed in the experiments suggest that the radical production depends on the irradiation temperature.

**Figure 2:**
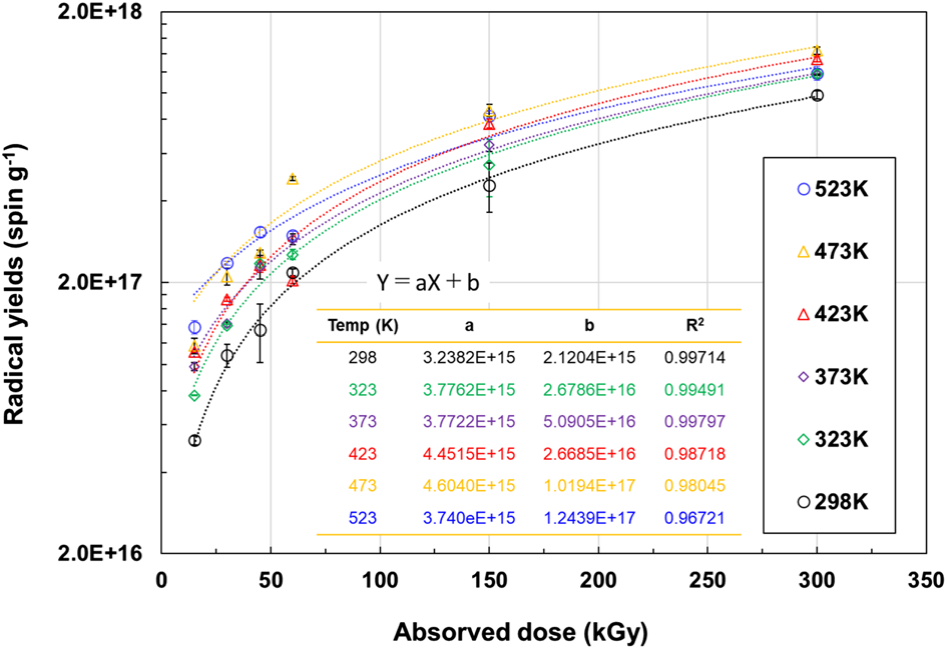
Radical yields as a function of absorbed dose. Irradiation carried out under nitrogen atmosphere, followed by exposure to air within 5 min and stored at 298 K under atmosphere until measurement. Curve fitting was performed by the least squares method, and the fitting parameters were summarized in in table.

Radical yields as a function of absorbed dose are shown in Fig. 2. Curve fitting was performed by the least squares method. Increasing the irradiation temperature results in higher radical yields in the low-dose range. The radical concentration increase at each irradiation temperature was almost proportional to the dose increase at the early stage, and then converged to a specific value. It was observed that the convergence point of the radical concentration was approximately 1.5 × 10^18^ spin g^−1^.

In present experimental system, 200 keV-EB is heterogeneously deposited the energy to the PTFE string. Especially, the overlapping of the spurs becomes large near the stopping range area, which is considered to have a large influence on the primary radical formation and stabilization. Moreover, the radiolysis of the PTFE is accompanied by formation the low molecular weight fragments/products (F·, F_2_ and CF_4_, etc.) of degradation the main chains. These species can react with induced radicals lowering their common concentration. Hence, the induced initial radicals undergo these primary process, and finally stable radicals are observed at 297 K by ESR. Furthermore, the influence of temperature might be explained as role of accelerating the mobility of ions as well as gaseous products.

Although the overlapping of spurs is taking place at early stage by EB irradiation because of high dose rate (5 kGy s^−1^)^21^, in the region where the radical yields are proportional to the dose, the residual radicals trapped at 297 K in PTFE are far from the neighboring radicals. This means that the radical density is low. Therefore, radical annihilation would hardly occur and the yield of each sample would be about 100%. It can be seen that the radical yields begin to be saturated by irradiation of 45 kGy or more. In the high-dose range where the radical yields are saturated, the radicals are annihilated because of the denser population of the radicals. As a result, the radical yields are reduced. Radical yields of some high temperature irradiated samples at 60 kGy were observed to be lower than 45 kGy. It is considered that this is because the radicals annihilated due to the overlap of the induced radicals, and as a result, the observation was lower. Since the radical yields at the higher dose stabilize regardless of irradiation temperature, the total amount of radicals that can be trapped in the crystalline PTFE state is approximately 1.5 × 10^18^ spin g^−1^.

**Figure 3:**
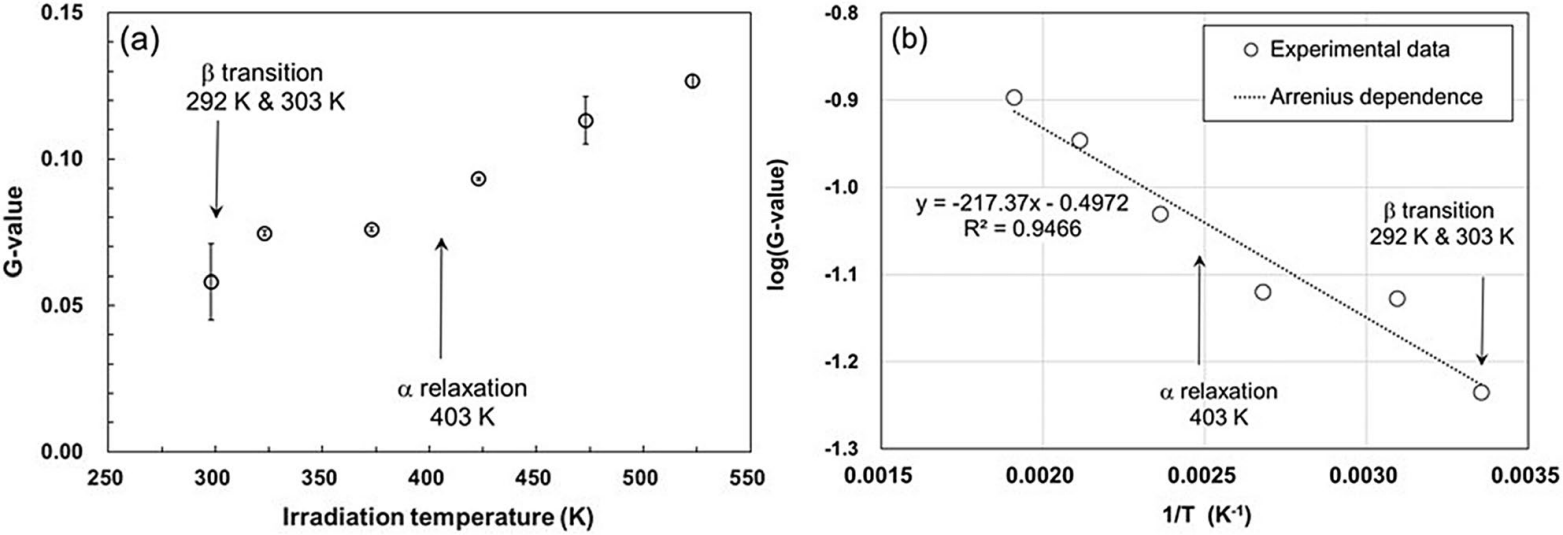
G(R·) as function of irradiation temperature. (**a**) G-value as a function of irradiation temperature. (**b**) Temperature dependence of the radiation-chemical yields of the formation of peroxide macroradicals in PTFE irradiated with 200-keV electrons, in the coordinates of the Arrhenius equation. G(R·) was calculated from the data in Fig. 2 at the proportional region (15–45 kGy) to the dose.

The G-values of trapped radicals (G(R·): number of radicals per 100 eV absorption energy) at each irradiation temperature were calculated from the data in Fig. 2 at the proportional region (15–45 kGy) to the dose, and plotted against the irradiation temperatures in Fig. 3a. In addition, Arrhenius plot was performed, as shown in Fig. 3b, and the activation energy was obtained from the slope. The value of the activation energy turned out to be extremely low about 1.81 kJ mol^−1^. G(R·) can be considered as the generation efficiency of the trapped radical. G(R•) determined during the irradiation at 297 K was 0.058 and G(R·) increased with increasing irradiation temperature corresponding to each relaxation and transition temperature. In particular, G(R·) increased noticeably above the α relaxation temperature (403 K). The α relaxation is the transition in the molecular motion of inter-polymer chains among crystallites^22^. According to a study which described the G(R·) of PTFE irradiated by γ-rays at RT using ESR^17,23,24^, G(R·) ranges approximately between 0.10–0.18. The G(R·) of our EB irradiation at 297 K was 0.058, which is lower than that of the γ-ray irradiation already reported. It is considered that there are two reasons. One is that there is to be irradiated/ non-irradiation area on the PTFE of 1 mm diameter by penetration depth of 200 keV-EB. The other one is that there may be differences in spatial distribution of spurs between the γ-rays and the low energy EB.

The penetration depth of 200 keV-EB with a specific gravity of 0.85 is approximately 500 μm, which means that radicals are heterogeneously induced in the sample. Furthermore, in electron stopping range area, it is possible that the effect of overlapping of spur becomes large and the radical generation is suppressed. From the study of modelling degradation of PTFE under electron irradiation^25^, it is reported that a large yield of secondary electrons at around 500 eV, and the spatial distribution of these electrons is about 15 nm. The 200 keV electrons thermalized in the PTFE to around 500 eV by electron scattering form a multi-spur which is overlapping spurs in stopping range area. It is suggested that the intermediate species formed in multi-spur are inactivated by ion-radical, radical–radical, Cage effect, etc. and suppress the production of trapped radicals.

The calculation predicted that the energy deposition of EB is about 40–50% of the entire PTFE with a diameter of 1 mm. As a result, G(R·) becomes lower than the literature value. If the obtained G(R·) is corrected with the effective EB penetration depth, it will be within the range of literature values (net G(R·) are predicted to be approximately 0.11–0.15 by correction of energy deposition, see Supplementary Fig. S1).

Furthermore, the G(R·) of PTFE irradiated at 77 K was reported to be 0.38^26^. At the cryogenic temperature (77 K), where the molecular motion of the polymer is frozen, it is considered that free radicals are not attenuated and all induced radicals are trapped after irradiation at 77 K. It has to be noted that G(R·) obtained by irradiation at RT is the value of radicals that remain trapped in crystalline, amorphous, or interface states, after the unstable radical is annihilated by molecular motion. Thus, G(R·) at 77 K is the net amount stably trapped in the initial reaction, regardless of temperature. The initial radiative yield (log [G_i_]) obtained from Arrhenius equation in Fig. 3b is − 0.497, which is good agreement of − 0.420 of the radiation-chemical yield of active centers at 77 K (log [G_77_]).

PTFE fine powder has a crystallinity of 93%. The trapped radicals gradually decay, even when stored for a long time at 297 K^18^. The decay of radicals is accelerated by heat treatment (see Supplementary Fig. S2), while the radicals will finally remain in the crystal lattice. This means that the radicals are trapped not only in the crystalline region but also in the para-crystalline region, or at the interfaces and inter-chain regions of the crystallites. Although the radical decay at higher temperatures is faster, the above experimental results show that the radical yields after irradiation at higher temperatures were higher than the irradiation at lower temperatures when compared at the same dose in the low-dose region. This suggests that the initial radical yield generated in PTFE differs depending on the irradiation temperature.

**Figure 4:**
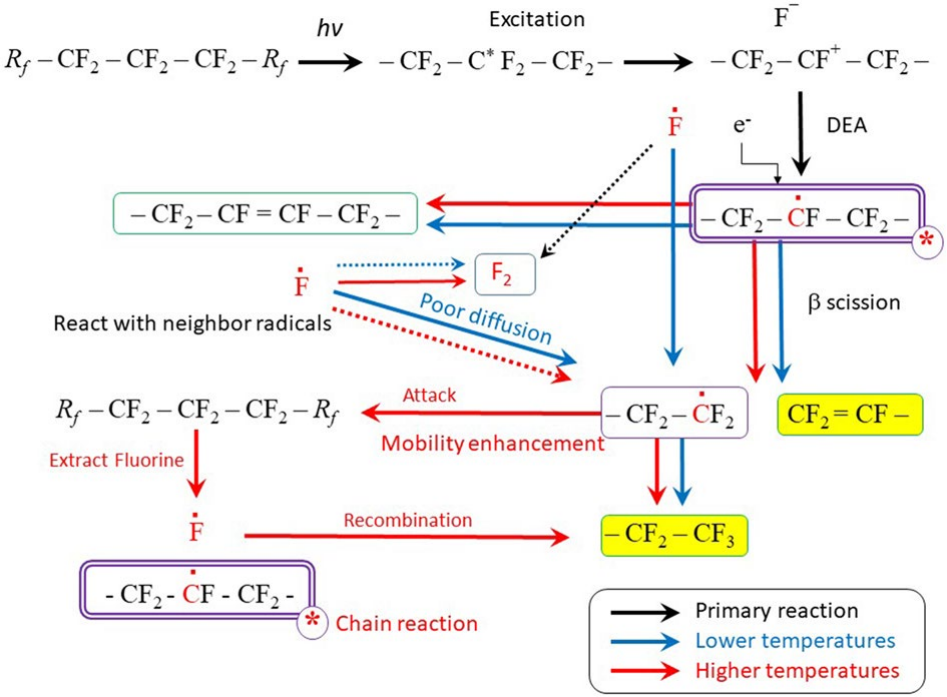
Reaction mechanism of PTFE irradiated at RT and higher temperatures. Solid line: main reaction, dotted line: minor reaction.

From a study of changes in the chain scission efficiency in the range below the Tm of PTFE performed by changes in the molecular weight using DSC^8^ it was reported that the higher the irradiation temperature, the higher the scission efficiency based on the C–C bond breakage. For example, scission efficiency of irradiated at 473 K is 3 times higher than 273 K irradiation. Although these phenomena are explained by the synergistic thermal and radiation effects^8^, the initial radiation-induced radical formation process in PTFE crystallites might be different because the obtained radical yields vary depending on the irradiation temperature. That is, dissociative electron attachment (DEA) may have temperature dependence, or the chemical reaction after DEA may have temperature dependence. Although the mechanism of DEA in condensed phase is not clear in detail up till now^25,27^, the formation process of macroradicals in PTFE irradiated under mutual action of electrons and temperature, cannot be explained by the DEA mechanism alone. The formation process of macroradicals is conventionally explained by DEA process (electron accepting fluorine atoms capture the electrons with high efficiency and dissociate to F^−^ ions). That is, when PTFE is irradiated under an oxygen-free atmosphere, fluoroalkyl radicals are induced by DEA. Moreover, the main chain scission, which is induced by fluoroalkyl radicals, occurs with the β-scission producing *R*_*f*_* − *CF_3_, *R*_*f*_ − CF = CF_2_ and an end-chain radical (see Fig. 4).

It has been experimentally suggested that DEA completes its reaction with a reaction time of picoseconds or less using picoseconds pulse radiolysis^28^. In present study, it cannot be concluded whether the DEA of the primary reaction is temperature dependence because of the product analysis by ESR. Since DEA is a physicochemical reaction and is conventionally considered independent of temperature, the net amount of initially produced radicals, which is equivalent to the amount of radicals at 77 K irradiation, are considered to be constant regardless of the irradiation temperature. These results indicate that the β-scission efficiency after the initially induced fluoroalkyl radical formation due to DEA depends on the molecular motion induced by the irradiation temperature. In fact, as the irradiation temperature increases, the chain scission is accelerated and the number of end-chains increases, as reported in a previous paper^8^. With respect to the radical yields induced by the chemical reaction after DEA, irradiation at higher temperatures improves the radical yields and a larger number of chain scission are observed compared to RT. This indicates that the radiation-induced chemical reaction increases with irradiation temperature.

**Figure 5:**
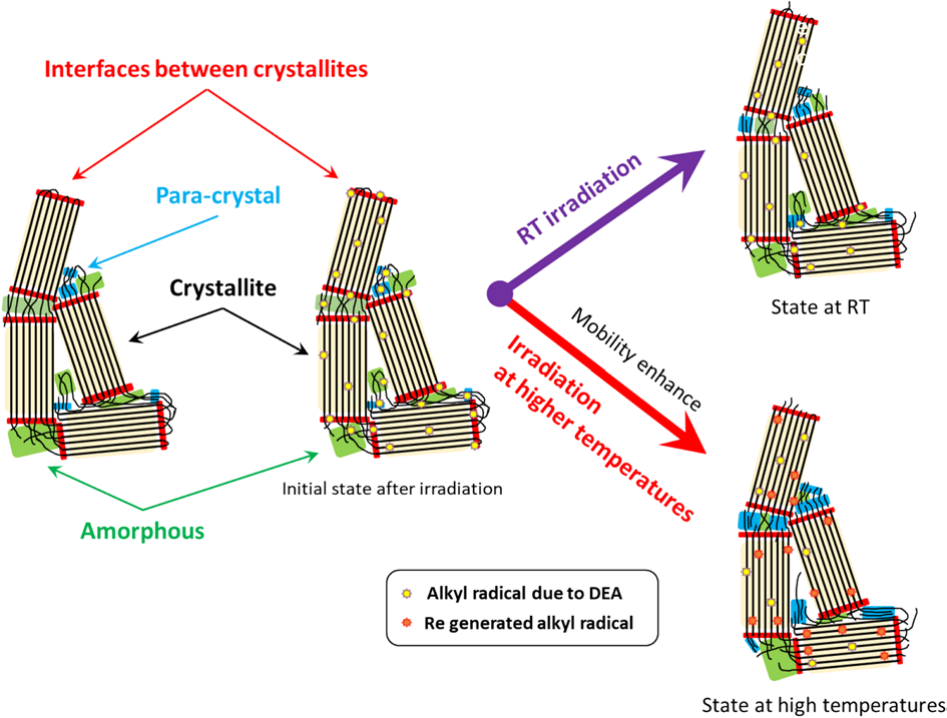
Scheme of radical trapping sites at RT and higher temperatures.

Because the obtained ESR spectrum (see Fig. 1) at each temperature is asymmetric, the radicals trapped in PTFE are mainly middle-chain type peroxide radicals trapped in the crystalline and para-crystalline region, or at the interfaces and inter-chain region of the crystallites (see Fig. 5). When heat treatment is applied, the radicals decay by molecular motion reducing the yields. In particular, the chain-end radicals easily degrade due to the higher molecular motion^18^. In contrast, in a crystallite lattice, radicals hardly decay because of the low molecular motion and are relatively stable.

The reason why the amount of radical G(R·) is higher at increased irradiation temperatures is illustrated by the reaction shown in Fig. 4. Energy deposition is randomly performed regardless of whether it is crystalline or amorphous, and then fluoroalkyl radicals are induced by DEA. With respect to the RT irradiation, fluoroalkyl radicals produced in the amorphous or para-crystalline region, and those at the interfaces between crystalline regions are less stable than those in the crystalline phase. Some fluoroalkyl radicals decay and form a double bond (*R*_*f*_ − CF = CF − *R*_*f*_) in the main chain of the polymer, while β-scission produces *R*_*f*_ − CF = CF_2_ with end-chain radicals. The end-chain radical reacts with the fluorine radical (F·) induced by DEA (difficult to diffuse due to low molecular motion in the system) or the F· radical generated at the same time the double bond is formed in the main chain. As a result, polymer chain-ends (*R*_*f*_ − CF_3_) are created reducing the radical yield.

As the molecular motion is enhanced, the diffusion of F· radicals induced by DEA is accelerated, enabling F· radicals to recombine more easily with neighboring F· radicals to form F_2_ gas, which then diffuses out of the matrix. Fluoroalkyl radicals might be produced by F_2_ gas during the diffusion. In addition, the end-chain radicals with higher molecular mobility react with the fluorine atoms on the main chain of PTFE, including those existing in the crystallites, forming end-chain and fluoroalkyl radicals^17^. When the regenerated fluoroalkyl radical is trapped in an unstable region such as the amorphous, para-crystalline, or interface regions, the same chain reaction repeatedly occurs resulting in a gradual radical decay. When fluoroalkyl radicals are regenerated in the relatively stable crystalline region, they remain trapped without contributing to the chain reaction. As a result, the number of induced scissions and the decrease in radical yields were not as high as compared to the irradiation at RT. That is, the G(R·) at higher irradiation temperature approaches the G(R·) value at 77 K (0.38)^26^, and the radical is trapped in the crystalline phase.

In condensed phase, electrons may exist in two states: (a) localized state, (b) quasi-free state corresponding to delocalized electrons which can be described as quasi-particle. The equilibrium between these ones may depend on the PTFE structure, phase state and the temperature, as already reported^29^. It suggests that the impact of the factors of structure and phase state can determine the synergistic nature of the observed thermal and radiation effects in PTFE. That is, the observed increase of the radical concentration with the elevated irradiation temperature may be connected with the change of the equilibrium conditions due to radiation-induced changes in polymer structure and phase state as well as the irradiation temperature.

Therefore, it can be concluded that the chain reaction by side-chain fluorine extraction, which is based on the end-chain radical generation induced via β-scission after DEA, is enhanced by synergistic thermal and radiation effects.

The low molecular weight (Mw) PTFE micropowder can be obtained by performing irradiation at ambient condition (297 K, air)^30^. However, it is reported that the irradiated PTFE contains a lot of end-chain radicals, so it produces perfluoroalkyl carboxylic acids (PFCAs) with high environmental load^18^. The obtained experimental results suggest that the high temperature irradiation suppresses the generation of PFCAs and also promotes the chain reaction of main chain scission. Therefore, "large suppression of by-products generate that are subject to environmental regulations" and "3 times higher or more efficiency of manufacturing of PTFE micropowder" are able to achieve. The high temperature irradiation below melting temperature for PTFE are a possibility to realize the precision polymer fabrication processing with low environmental load and high volume mass productivity.

## Conclusion

The behavior of trapped free radicals in highly crystalline PTFE after EB irradiation at various temperatures under a nitrogen atmosphere was studied by ESR. G(R·) increases with increasing irradiation temperature corresponding to each of the relaxation and transition temperatures. It is concluded that the chain reaction by fluorine extraction from the main chain, induced by end-chain radicals generated via β-scission after DEA, is enhanced by synergistic thermal and radiation effects.

## Supplementary Information

Supplementary Information.
