# Supplementary material for: Trapped radical behavior of electron beam irradiated polytetrafluoroethylene fine powder at various temperatures
Source: Sci Rep. 2021 May 25;11:10907. doi: 10.1038/s41598-021-90462-6 (PMC8149384; doi:10.1038/s41598-021-90462-6)
Supplement: Supplementary file 1 — Supplementary Information. [file 41598_2021_90462_MOESM1_ESM.docx]

Supplementary Information

Trapped radical behavior of electron beam irradiated polytetrafluoroethylene fine powder at various temperatures

Akihiro Oshima^1,2^, Hiroto Horiuchi^3^, Atsushi Nakamura^3^, Shun Kobayashi^3^,

Ayana Terui^3^, Ayano Mino^3^, Ryoya Shimura^3^, Masakazu Washio^3^

^1^ Graduate School of Engineering, Osaka University, 2-1 Yamadaoka, Suita, Osaka, 565-0871, Japan

^2^ The Institute of Scientific and Industrial Research, Osaka University, 8-1 Mihogaoka, Ibaraki, Osaka, 567-0047, Japan

^3^ Waseda Research Institute for Science and Engineering, Waseda University, 3-4-1, Okubo, Shinjuku, Tokyo, 169-8555, Japan

*Correspondence to: Akihiro Oshima, Graduate School of Engineering, Osaka University, 2-1 Yamadaoka, Suita, Osaka, 565-0871, Japan, e-mail: akoshima@sanken.osaka-u.ac.jp

**This file includes:**

Materials and Methods

Materials

Irradiation vessel

Dosimetry

Figure S1

Figure S2

**Materials and Methods**

**Materials**

The PTFE string was obtained by mixing oil with fine powder, and then molding it with an extruder, and volatilizing the oil to obtain a string with a diameter of 1 mm. At that time, the shape of the beginning region and the end region of the molding are non-uniform, and the apparent density is significantly different, so they are excluded in the experiment.

**Irradiation vessel**

An external shape of irradiation vessel with a heating device is 210^L^×210^W^×53^T^ mm^3^. Due to the specifications of the EB equipment, the effective irradiation area is 15 cm square, but due to the temperature distribution of the hot plate type heating device (maximum temperature: 723 K), 14 cm square is the effective irradiation area in this experiment. The uncertainty of temperature is obtained by dividing the irradiation area (14 cm square) in the irradiation vessel into 9 region, measuring 9 points at the center of divided area with a K-type thermocouple, and measuring the distribution. As a result, there is a 5 K uncertainty at the desired sample temperature.

**Dosimetry**

Dosimetry was performed using a CTA (cellulose triacetate) dosimeter (FUJIFILM, FTR-125). The dosimeter was placed in an irradiation vessel, and 4 pass EB irradiation (electron energy: 200 keV and electron current: 1.0 mA, stage transfer speed: 2.0 m min^-1^, work distance: 47 mm, effective irradiation area: 20^L^ × 10^W^ cm^2^) was performed. And then the absorbed dose per pass when irradiated with an electron current of 0.5 mA was obtained at irradiation point. The dose rate was 5 kGy s^-1^ which was calculated from the effective irradiation area, the dose per pass and stage transfer speed.

**Figures**

**
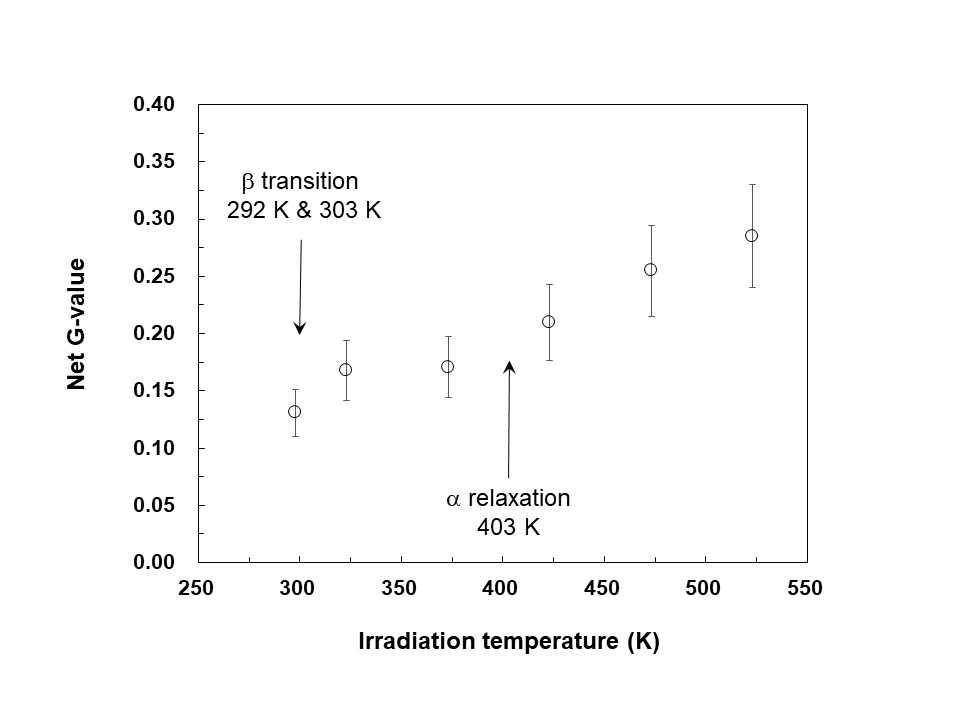
**

**Figure S1.** Net G(R•) as a function of irradiation temperatures. Net (R•) was estimated by penetration range of EB according to the Monte Carlo simulation code.

**
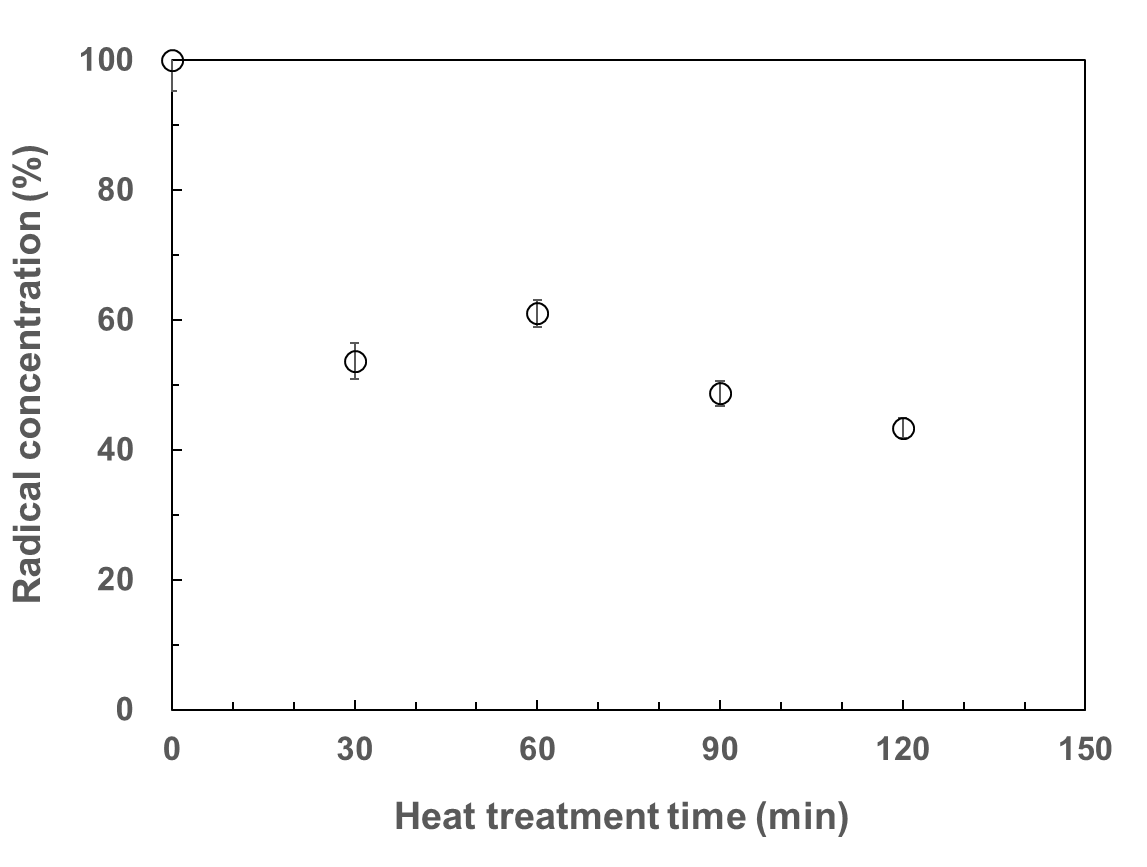
**

**Figure S2.** Radical concentration as a function of heat treatment time. Irradiation was carried out at 298 K with a dose of 60 kGy under nitrogen followed by exposure to air at 298 K within 5 minutes (radical yields = 2.04 × 10^17^ spin g^-1^). The thermal treatment was conducted at 473 K under nitrogen atmosphere.
